# Laboratory studies evaluating the efficacy of a novel orally administered combination product containing sarolaner, moxidectin and pyrantel (Simparica Trio™) for the treatment and control of flea infestations on dogs

**DOI:** 10.1186/s13071-020-3944-3

**Published:** 2020-03-01

**Authors:** Kristina Kryda, Sean P. Mahabir, Lori Carter, William R. Everett, David R. Young, Leon Meyer, Mirjan Thys, Sara Chapin, Susan J. Holzmer, Csilla Becskei

**Affiliations:** 1 Zoetis, Veterinary Medicine Research and Development, 333 Portage Street, Kalamazoo, MI 49007 USA; 2 Stillmeadow, Inc., 12852 Park One Drive, Sugar Land, TX 77478 USA; 3 BerTek, Inc., PO Box 606, Greenbrier, AR 72058 USA; 4 Young Veterinary Research Services, Inc. (YVRS), 7243 East Ave, Turlock, CA 95380 USA; 5 Clinvet International (Pty) Ltd, Uitsigweg, Bainsvlei, Bloemfontein, 9338 Republic of South Africa; 6 Zoetis, Veterinary Medicine Research and Development, Mercuriusstraat 20, 1930 Zaventem, Belgium

**Keywords:** *Ctenocephalides canis*, *Ctenocephalides felis*, Oral combination, Speed of kill

## Abstract

**Background:**

Five studies were conducted to evaluate a novel oral combination tablet containing sarolaner, moxidectin and pyrantel (Simparica Trio™), for efficacy against induced flea infestations, speed of kill and effects on flea reproduction on dogs.

**Methods:**

Based on pre-treatment flea counts, dogs were randomly allocated to treatment with a single, oral dose of either placebo or Simparica Trio™ at the minimum label dose of 1.2 mg/kg sarolaner, 24 µg/kg moxidectin and 5 mg/kg pyrantel (as pamoate salt) on Day 0. All dogs were infested with approximately 100 unfed, adult fleas (*C. felis* or *C. canis*) prior to treatment and weekly for 5 weeks post-treatment. In Studies 1, 2 and 3, the number of viable fleas were comb-counted at 24 h after treatment and after each weekly infestation; Study 2 also included groups treated with tablets containing sarolaner-alone (1.2 mg/kg), moxidectin-alone (24 µg/kg) or pyrantel-alone (5 mg/kg). In Study 4, flea counts were conducted at 3, 4, 8 and 12 h after treatment and subsequent weekly infestations to establish speed of kill. In Study 5 (flea reproduction), dogs were housed in an enclosure designed to facilitate collection of flea eggs.

**Results:**

Efficacy of Simparica Trio™ against *C. felis* was ≥ 99.7% and against *C. canis* was 100% at 24 h after treatment and after subsequent infestations for at least 35 days. Treatment with sarolaner-alone had similar efficacy to Simparica Trio™, while moxidectin-alone and pyrantel-alone were no different from placebo at most time points. In Study 4, significant flea killing started at 4 h after treatment; by 8 h after treatment, all treated dogs were free of fleas. Following weekly re-infestation, the combination product reduced fleas by ≥ 97.8% within 12 h for 28 days. Simparica Trio™ reduced flea egg-laying by 100% for 35 days. No treatment-related adverse reactions occurred in any study.

**Conclusions:**

A single dose of Simparica Trio™ at the recommended minimum dose provided highly efficacious and rapid treatment within 4 h of existing flea infestations and persistent control of fleas on dogs for 5 weeks. The efficacy against fleas resulted in 100% prevention of flea reproduction for over a month following a single oral dose.

## Background

Dogs can be affected by numerous internal and external parasites which can have direct deleterious effects on their hosts and potentially transmit disease agents to dogs and humans [1, 2]. Of the most common parasites, fleas frequently infest dogs worldwide [3] with the cat flea (*Ctenocephalides felis felis*) being the most common species followed by the dog flea (*Ctenocephalides canis*). Flea infestations are very common in dogs but often go unnoticed by pet owners [4] unless the dog develops pruritus or the flea infestation becomes severe, in which case newly emerged fleas may also jump on and bite the owner. Fleas cause irritation due to their direct blood-feeding activity and heavy flea infestations may lead to anemia, especially in immature animals [5]. Dogs and cats can be highly sensitive to flea bites and the development of flea allergy dermatitis (FAD) is common [6, 7]. Fleas can transmit zoonotic pathogens such as *Rickettsia felis* [8], *Rickettsia typhi* [9] and *Bartonella henselae* [10, 11], and are the intermediate hosts for the tapeworm *Dipylidium caninum* [3]. Due to the ubiquitous nature of fleas, their ability to potentially induce clinical signs such as pruritus, and the possibility of transmitting diseases to the dog including zoonotic disease agents, efficacious treatment and control of flea infestations is a significant concern for pet owners and veterinarians [12]. To alleviate the direct negative impact of fleas and to reduce the risks of disease transmission, year-round flea control should therefore be considered for pets in most geographical areas [3, 13].

Sarolaner is an isoxazoline, a potent new class of ectoparasiticides in companion animals that provides broad activity against fleas and ticks [14]. Sarolaner inhibits the function of the neurotransmitter gamma aminobutyric acid (GABA) receptor and glutamate receptor, acting at the neuromuscular junction in insects which results in uncontrolled neuromuscular activity leading to death in fleas and ticks [14, 15]. Sarolaner is rapidly absorbed following oral administration with C_max_ occurring within the first day post dose and a half-life of 12 days [14]. Recently, a novel oral combination product that contains sarolaner in addition to moxidectin and pyrantel (Simparica Trio™, Zoetis, Parsipanny, NJ, USA) has been developed to provide not only treatment and control of flea and tick infestations for 1 month in dogs but also treatment of roundworm and hookworm infections and protection from heartworm and lungworm disease.

In this paper, we report a series of laboratory studies that evaluated the efficacy of Simparica Trio™ at a minimum label dose of 1.2 mg/kg sarolaner, 24 µg/kg moxidectin and 5 mg/kg pyrantel (as pamoate salt) against the most common flea species, *C. felis* and *C. canis*, infesting dogs. The studies include direct evaluation of treatment and prevention of flea infestations for 5 weeks after a single dose, flea speed of kill and effects on flea reproduction.

## Methods

Five placebo-controlled, masked and randomized studies were conducted, including three dose confirmation studies. Studies 1 and 2 evaluated efficacy against the cat flea, *C. felis*. Study 2 included investigation of non-interference of the individual active components in the combination product and Study 3 measured efficacy against the dog flea, *C. canis*. Study 4 evaluated speed of kill against *C. felis* and Study 5 investigated the effects of the treatment on flea reproduction. The studies were conducted in accordance with the World Association for the Advancement of Veterinary Parasitology (WAAVP) guidelines for evaluating the efficacy of parasiticides for the treatment, prevention and control of flea and tick infestation on dogs and cats [16] and complied with Good Clinical Practice and VICH guideline GL9 [17]. Masking was accomplished by separation of functions of study personnel. All personnel conducting study observations were unaware of treatment assignments and dedicated personnel conducted the allocation of dogs and treatment dispensing but did not conduct any other study observations.

### Animals

In Studies 1, 2, 3 and 4, 8 dogs were allocated per treatment group for each study in order to provide > 95% power to show both statistical significance and at least 90% reduction compared with placebo for each post-treatment flea count. This assumed a control mean flea count of 20–80 for the placebo. In Study 5, 10 dogs were allocated per treatment group in order to provide > 90% power to show both statistical significance and at least 90% reduction compared to placebo for each flea egg count and number of larvae and adults emerged from incubated eggs. This assumed a control mean egg count of at least 100 for the placebo. Dogs were deemed to be in good health by a veterinarian at enrollment and had undergone a wash-out period determined sufficient by the investigator to ensure that no residual efficacy remained from any previously administered pulicidal compounds. Additionally, the adequacy of the wash-out period was confirmed *via* host suitability flea infestations and counts that were performed prior to study start which ensured that selected dogs could maintain an adequate infestation. Dogs were housed individually in enclosures that prevented any physical contact between animals and conformed to accepted animal welfare guidelines. For acclimatization, dogs were brought into the study site facilities for at least a week prior to treatment. Dogs were fed an appropriate maintenance amount of a commercial food and water was available *ad libitum* for the duration of the study.

#### Dose confirmation studies

The studies used adult purpose-bred, Beagles and mixed breed dogs of both sexes, ranging in age from 6 months to 7 years and weighing from 6.1 to 25.0 kg. Sixteen dogs were used in each of Studies 1 and 3, and 40 dogs were included in Study 2, as this non-interference study also included groups treated with individual components of the combination product.

#### Speed of kill study

Sixty-four Beagle and mixed breed dogs of both sexes, ranging in age from 10 months to 8 years and weighing from 6.5 to 15.7 kg were used in Study 4.

#### Flea reproduction study

Twenty Beagles of both sexes, ranging in age from 3.6 to 4 years and weighing from 9.0 to 13.5 kg were used in Study 5.

### Design

General health of all of the dogs was observed at least twice daily for the duration of the studies. To determine host suitability, dogs were infested with approximately 100 fleas prior to study start. The fleas were removed and counted 24 h after infestations. From a pool of dogs, dogs with the highest host suitability live flea counts were chosen for inclusion in each study. The dogs were blocked by host suitability flea counts and then assigned to treatment groups in a randomised complete block design.

#### Dose confirmation studies

In Studies 1 and 3, one placebo-treated group and one combination product-treated group were enrolled (*n* = 8 per group). Study 2 included five treatment groups (*n* = 8 per group): placebo; combination product; sarolaner-alone; moxidectin-alone; and pyrantel-alone.

#### Speed of kill study

Four separate pairs of placebo-treated and combination product-treated groups were used (*n* = 8 per group).

#### Flea reproduction study

One placebo-treated group and one combination product-treated group were enrolled (*n* = 10 per group).

### Treatment

In Studies 1, 3, 4 and 5, dogs were dosed on Day 0 with either placebo tablets containing inert formulation ingredients (vehicle) or combination product tablets and in Study 2 additional groups were dosed with tablets containing the individual formulation components of the combination product (sarolaner-alone, moxidectin-alone or pyrantel-alone). Placebo and active tablets were similar in presentations to maintain masking. Tablets of varying strengths were provided, such that a combination of tablets could be administered to ensure dogs were appropriately dosed to the minimum end of the proposed label dose range. Each dog received a single or a combination of different tablet strengths containing all three active ingredients (or in Study 2 the individual formulation components) to provide as close as possible to the minimum label dosage of 1.2 mg/kg sarolaner (actual doses ranged from 1.2 to 1.6 mg/kg), 24 µg/kg moxidectin (actual doses ranged from 24 to 32 µg/kg) and 5 mg/kg pyrantel (as pamoate salt) (actual doses ranged from 5.0 to 6.6 mg/kg) or the equivalent number of placebo tablets based on pre-treatment body weights. Feed was withheld for at least 12 h prior to treatment and animals were not fed again until at least 4 h post-treatment. All doses were administered by hand pilling to ensure complete dosing. Each dog was observed for several minutes after dosing for evidence that the dose was swallowed and for up to 2 h post-dosing for any signs of emesis. Dogs were examined for general health and any reactions to treatment at 1, 3, 6 and 24 h after treatment.

### Flea infestations

Infestations were performed by applying the fleas directly to the fur while the dogs were restrained for a few minutes until the fleas dispersed into the hair coat. Approximately 100 unfed viable adult *C. felis* (*C. canis* in Study 3) were applied at each infestation to each dog.

#### Dose confirmation studies

Each dog was infested with fleas on Days-1, 6, 13, 20, 27 and 34. The fleas in Study 1 originated from a *C. felis* colony that had been initiated with fleas from Germany 6 years prior to the start of the study; new field-collected fleas from Ireland had been introduced to this colony 4 years prior to the study. The fleas in Study 2 originated from a *C. felis* colony that had been initiated with fleas from Kansas State University, Manhattan, Kansas, USA, to which additional fleas were periodically introduced from EL Labs, Soquel, California, USA, with the last introduction of new fleas occurring about 3 months prior to the study. The *C. canis* used in Study 3 originated from a colony that had been initiated with dog fleas collected from dogs in Ireland approximately 7 years prior to the study.

#### Speed of kill study

The dogs were infested with fleas on Days-1, 7, 14, 21, 28 and 35. The fleas originated from a *C. felis* colony which had been initially obtained from a laboratory colony in North Carolina, USA, enriched with wild caught fleas from Arkansas, USA, approximately 3 years prior to the study.

#### Flea reproduction study

The dogs were infested with fleas on Days-1, 5, 12, 19, 26 and 33. The fleas originated from a *C. felis* colony that had been initiated with fleas caught from naturally infested animals in California, USA. The colony was periodically enriched with locally-sourced wild fleas, with the last introduction occurring approximately 4 months prior to the study.

### Flea counts and evaluation of reproduction

Flea counts were conducted by systematically combing the coat of each dog with a fine-toothed flea comb for at least 10 min to remove and count live fleas. Any dog on which fleas were found in the last 5 min (1 min in Studies 2 and 5) was combed for an additional 5 min (1 min in Studies 2 and 5) and this process was continued until no fleas were recovered during the final combing period. Protective clothing, flea combs and gloves were changed between animals.

#### Dose confirmation studies

Flea counts were conducted 24 h after treatment and after weekly re-infestations.

#### Speed of kill study

Flea counts were conducted 3, 4, 8 or 12 h after treatment and after each weekly re-infestation for the four separate pairs of placebo-treated and combination product-treated groups.

#### Flea reproduction study

At 24 h after treatment and 48 h after each post-treatment re-infestation, each dog was held for 20 h in an enclosure specially designed to facilitate the collection of flea eggs. At the end of this period, adult flea counts were conducted on each dog and the fleas were removed from the dogs. For the flea egg collection, the animal’s fur was ruffled by hand to dislodge any eggs retained in the coat and then all eggs were collected from the tray beneath the enclosure. For each dog, all eggs were counted and up to 100 randomly selected flea eggs were transferred to a container with an appropriate growth medium and maintained in an incubator under the appropriate conditions for egg hatching. After 5 days, the emerged viable larvae were counted. A further sample of up to 100 randomly selected flea eggs from each dog was transferred to a container with growth medium and maintained in an incubator under appropriate conditions for flea development, the emerged adult fleas being counted after 35 days.

### Statistical analysis

Arithmetic means were used to summarize flea counts by treatment and day of study. Flea counts were analysed using a general linear mixed model for each day of study (SAS Release 9.4, SAS Institute Inc., Cary, North Carolina, USA). The model included the fixed effect of treatment and random effects of block and error. If multiple rooms were used the random effects included room, block within room, and error. Hypothesis testing was two-sided at the significance level of α = 0.05.

For adult flea, flea egg and flea larva counts, percent reduction relative to the placebo group (efficacy) was calculated using the formula: [(C − T)/C] × 100, where C is the mean count for the placebo group and T is the mean count for the treated group.

## Results

### Dose confirmation studies

Placebo-treated dogs consistently maintained flea infestations in all three dose confirmation studies. In the two studies with *C. felis*, arithmetic mean live flea counts for placebo-treated dogs ranged between 78.5–89.5 in Study 1 (Table 1) and 54.1–86.6 in Study 2 (Table 2). For *C. canis* mean counts for placebo dogs ranged between 74.8–87.9 in Study 3 (Table 1). Efficacy of the combination product against *C. felis* was ≥ 99.9% against existing infestations and at least 99.7% against subsequent infestations for 35 days after single treatment in both studies. For *C. canis*, efficacy was 100% against both existing infestations and against subsequent infestations for 35 days after a single treatment. Flea counts for the combination product were significantly lower (7.78 ≤ *t*_*df*_ ≤ 34.43, 7 ≤ *df* ≤ 35, *P* < 0.0001) than the placebo at all counts in all three studies (Tables 1 and 2).

**Table 1:** Arithmetic mean flea counts and percent efficacy relative to placebo at 24 h after treatment and weekly re-infestations with *Ctenocephalides felis* or *C. canis* for dogs treated orally with Simparica Trio™

Day after treatment	Treatment group	Study 1 (*C. felis*)		Study 3 (*C. canis*)	
		Flea count	% Efficacy	Flea count	% Efficacy
1	Placebo	89.5		85.5	
	Simparica Trio™	0.1*	99.9	0*	100
7	Placebo	78.5		74.8	
	Simparica Trio™	0*	100	0*	100
14	Placebo	82.8		75.3	
	Simparica Trio™	0*	100	0*	100
21	Placebo	80.5		87.9	
	Simparica Trio™	0*	100	0*	100
28	Placebo	88.1		81.8	
	Simparica Trio™	0*	100	0*	100
35	Placebo	89.5		75.3	
	Simparica Trio™	0*	100	0*	100

*Flea counts are significantly lower than placebo; 14.45 ≤ *t*_*df*_ ≤ 34.43, 7 ≤ *df* ≤14, *P* < 0.0001

**Table 2:** Arithmetic mean flea counts and percent efficacy relative to placebo at 24 h after treatment and weekly re-infestations with *Ctenocephalides felis* for dogs treated orally with Simparica Trio™, sarolaner-only, moxidectin-only or pyrantel-only tablets (Study 2)

Day after treatment	Treatment group	Flea count	% Efficacy
1	Placebo	70.1	–
	Simparica Trio™	0*	100
	Sarolaner	0*	100
	Moxidectin	65.5	6.6
	Pyrantel	66.6	5.0
7	Placebo	78.3	–
	Simparica Trio™	0*	100
	Sarolaner	1.9*	97.6
	Moxidectin	66.4	15.2
	Pyrantel	73.8	5.8
14	Placebo	65.8	–
	Simparica Trio™	0*	100
	Sarolaner	0*	100
	Moxidectin	70.6	0
	Pyrantel	76.8	0
21	Placebo	54.1	–
	Simparica Trio™	0.1*	99.8
	Sarolaner	0.4*	99.3
	Moxidectin	64.8	0.0
	Pyrantel	71.8^a^	0.0
28	Placebo	86.6	–
	Simparica Trio™	0.3*	99.7
	Sarolaner	0*	100
	Moxidectin	78.9	8.9
	Pyrantel	63.5^b^	26.7
35	Placebo	79.6	–
	Simparica Trio™	0*	100
	Sarolaner	0.4*	99.5
	Moxidectin	79.4	0.3
	Pyrantel	87.8	0

*Flea counts are significantly lower than placebo; 7.78 ≤ *t*_(35)_ ≤ 10.42, *P* < 0.0001

^a^Flea count significantly higher than placebo; *t*_(35)_ = − 3.00, *P* = 0.005

^b^Flea count significantly lower than placebo; *t*_(35)_ = 2.78, *P* = 0.0087

In the non-interference evaluation (Study 2), treatment with sarolaner-alone resulted in 100% efficacy against existing infestations and efficacy of 97.6% or greater for at least 35 days after a single treatment. Flea counts for treated dogs were significantly lower (7.78 ≤ *t*_(35)_ ≤ 10.42, *P* < 0.0001) than placebo at all counts (Table 2). Both moxidectin-alone and pyrantel-alone had little or no efficacy against fleas (Table 2). For moxidectin, flea counts were no different to those for placebo-treated dogs for all counts (− 1.81 ≤ *t*_(35)_ ≤ 1.49, 0.0791 ≤ *P* ≤ 0.1442) with flea count reductions ranging from 0 to 15.2%. Similarly, for pyrantel, flea counts were no different to those for placebo-treated dogs on most counts (− 1.30 ≤ *t*_(35)_ ≤ 0.57, 0.2018 ≤ *P* ≤ 0.6854) with reductions ranging from 0 to 5.8%. On Day 21, the flea count for the pyrantel group was significantly higher than that for placebo (*t*_(35)_ = − 3.00, *P* = 0.005) and on Day 28, the flea count for pyrantel was significantly lower than that for placebo (*t*_(35)_ = 2.78, *P* = 0.0087) with a reduction of 26.7%.

### Speed of kill study

In the speed of kill study (Study 4), all placebo-treated groups consistently maintained flea infestations for the duration of the study, with arithmetic mean counts between 72.4–98.8 fleas (Table 3). A significant reduction in flea counts compared to placebo (*t*_(53)_ = 4.16, *P* = 0.0001) was first observed within 4 h after treatment and all treated dogs were free of fleas by 8 h after treatment. Following weekly re-infestation, the combination product significantly (2.94 ≤ *t*_*df*_ ≤ 19.46, 10.6 ≤ *df* ≤ 56, *P* ≤ 0.0138) reduced flea counts relative to placebo within 4 h after infestations up to and including Day 21 and within 8 h through Day 35. At 12 h after post-treatment re-infestations, efficacy was ≥ 97.8% for 4 weeks and was 85.6% on Day 35.

**Table 3:** Arithmetic mean flea counts (percent efficacy) relative to placebo for dogs treated orally with Simparica Trio™ at 3, 4, 8 or 12 h after treatment (Day 0) and weekly re-infestations with *Ctenocephalides felis* (Study 4)

Count time	Treatment group	Day after treatment					
		0	7	14	21	28	35
3 h	Placebo	77.1	95.0	89.0	93.8	90.4	98.8
	Simparica Trio™	77.8 (0)	61.6^b^ (35.1)	50.1^b^ (43.7)	88.3 (5.9)	92.8 (0)	93.0 (5.8)
4 h	Placebo	72.4	94.1	89.5	93.1	95.9	97.4
	Simparica Trio™	42.8^a^ (40.9)	43.6^b^ (53.7)	31.3^b^ (65.1)	72.1^b^ (22.6)	85.9 (10.4)	95.9 (1.5)
8 h	Placebo	84.0	88.9	89.1	87.8	94.0	94.1
	Simparica Trio™	0^b^ (100)	0.4^b^ (99.6)	6.9^b^ (92.3)	3.6^b^ (95.9)	26.6^b^ (71.7)	70.4^b^ (25.2)
12 h	Placebo	85.0	93.1	86.6	92.0	96.0	95.3
	Simparica Trio™	0^b^ (100)	0^b^ (100)	0^b^ (100)	0^b^ (100)	2.1^b^ (97.8)	13.8^b^ (85.6)

^a^Mean live flea counts significantly lower than placebo; *t*_(53)_ = 4.16, *P* = 0.0001

^b^Mean live flea counts significantly lower than placebo; 10.09 ≤ *t*_*df*_ ≤ 41.72, 7 ≤ *df* ≤ 56, *P* ≤ 0.0138

### Flea reproduction study

Placebo-treated dogs maintained flea infestations and fleas had good fecundity throughout Study 5. Arithmetic mean live flea counts ranged between 60.8–73.8 and arithmetic mean flea egg counts were between 364.0–496.2 (Table 4). Arithmetic mean flea egg hatch rates for fleas from placebo-treated dogs ranged from 55.0% to 70.9% and 69.0% to 77.2% of eggs completed development to adult fleas. No live fleas were recovered and no flea eggs were collected from any dog treated with the combination product and thus there were no flea eggs available to evaluate for hatch or development to adult fleas.

**Table 4:** Arithmetic mean flea and egg counts and percent efficacy relative to placebo after treatment and weekly re-infestations with *Ctenocephalides felis* for dogs treated orally with Simparica Trio™ (Study 5)

Day after treatment	Treatment group	Flea count	% Efficacy	Egg count	% Efficacy
1	Placebo	73.8		364.0	
	Simparica Trio™	0*	100	0	100
7	Placebo	71.7		444.5	
	Simparica Trio™	0*	100	0	100
14	Placebo	67.0		496.2	
	Simparica Trio™	0*	100	0	100
21	Placebo	61.5		488.5	
	Simparica Trio™	0*	100	0	100
28	Placebo	60.8		440.6	
	Simparica Trio™	0*	100	0	100
35	Placebo	63.6		366.8	
	Simparica Trio™	0*	100	0	100

*Flea counts are significantly lower than placebo; 10.09 ≤ *t*_(9)_ ≤ 41.72, *P* < 0.0001

### Health observations

No adverse events related to the treatment of the dogs with the combination product or single component oral tablets were noted in any of the studies. Two dogs in Study 4, one dosed with placebo the other with the combination product tablet, were noted to have a small amount of frothy yellow vomitus under their pens at 6 h post-dose. This was considered possibly related to the assisted administration of the tablets and resolved without treatment. The only other health observations were minor ailments such as dermatitis and otitis, typically expected in laboratory dogs exposed to repeated flea infestation.

## Discussion

The studies reported here confirm the high efficacy of Simparica Trio™ against flea infestations for over a month after a single oral treatment at the minimum recommended dose. Efficacy was confirmed in three studies against the two most common flea species found on dogs, *C. felis* and *C. canis*, with flea counts for existing infestations reduced by ≥ 99.9% within 24 h after treatment, and ≥ 99.7% efficacy within 24 h against subsequent re-infestations for up to 5 weeks after treatment. In the non-interference study, comparison with the individual active ingredients of the combination tablet confirmed that flea efficacy was due to the sarolaner; the sarolaner-alone tablet had similar efficacy to the combination product at all time points, while the single component moxidectin and pyrantel tablets were no different to placebo at most time points (− 1.81 ≤ *t*_(35)_ ≤ 2.78, 0.0791 ≤ *P* ≤ 0.6854). On Day 28, the flea count for the single component pyrantel tablets was significantly lower by 26.7% than placebo (*t*_(35)_ = 2.78, *P* = 0.0087) and on Day 21 it was significantly higher (*t*_(35)_ = − 3.00, *P* = 0.005). The speed of kill study showed that treatment with the combination product resulted in the rapid onset of activity against fleas with live flea counts significantly reduced by 4 h after treatment. For post-treatment re-infestations, counts were significantly reduced from 3 h after infestation on Days 7 and 14, from 4 hours on Day 21 and from 8 h on Days 28 and 35. By 8 h after treatment efficacy was 100% and following re-infestation efficacy was ≥ 92.3% through Day 21, 71.7% on Day 28 and 25.2% on Day 35. By 12 h after treatment or re-infestation efficacy was 100% through Day 21, 97.8% on Day 28 and 85.6% on Day 35.

A rapid speed of kill is an important characteristic of an effective pulicide, since it provides quick relief from the irritation of flea infestation which is especially critical for the management of FAD [18]. Additionally, providing efficacy early enough to break the life-cycle is critical to the control of infestations of the premises, killing fleas before they have a chance to lay eggs and re-infest households and pets [19]. The impact of the rapid speed of kill of Simparica Trio™ was demonstrated in the flea reproduction study where treatment resulted in the complete cessation of flea egg-laying, likely due to the direct efficacy against adult fleas. Thus, treatment completely halted the flea life-cycle.

Fleas are a year-round parasite of dogs throughout the world and regular parasiticidal treatment is needed to prevent infestations, ameliorate the irritation and debilitation of their blood-feeding, FAD, and reduce the potential risk for disease transmission [3]. Compliance by owners is best supported with simple, convenient, regular treatments that may be best achieved with combination broad-spectrum products. Flea control is best included in a management programme for the other major parasites of dogs that need regular treatment or prevention such as ticks, heartworm, lungworm and gastro-intestinal nematodes. Flea control with the combination product was rapid, highly effective, and persisted for at least a month following a single oral dose and also completely prevented flea reproduction. The combination of sarolaner, moxidectin and pyrantel in a single oral chewable tablet provides a convenient and highly effective monthly treatment that protects against fleas, as well as against other major parasites of dogs that can be given year-round.

## Conclusions

A single oral dose of the combination product (Simparica Trio™) administered at the recommended minimum label dose of 1.2 mg/kg sarolaner, 24 µg/kg moxidectin and 5 mg/kg pyrantel (as pamoate salt) was highly effective and provided rapid treatment of existing flea infestations and continuous control of fleas on dogs for a month. Both the cat flea (*C. felis*) and the dog flea (*C. canis*) were effectively controlled. Sarolaner was confirmed as the component providing the treatment and control of fleas. A single treatment started to kill the existing flea infestation within 4 h and subsequent re-infestations within 8 h for at least one month. Fleas were killed rapidly and resulted in the complete cessation of flea reproduction for over a month following a single oral dose.

## Data Availability

Data upon which the conclusions are based have been presented in the article.

## Abbreviations

FAD: Flea allergy dermatitis
GABA: Gamma aminobutyric acid
VICH: International Cooperation on Harmonization of Technical Requirements for Registration of Veterinary Medicinal Products
WAAVP: World Association for the Advancement of Veterinary Parasitology
